# Specific protein 1(SP1) regulates the epithelial-mesenchymal transition via lysyl oxidase-like 2(LOXL2) in pancreatic ductal adenocarcinoma

**DOI:** 10.1038/s41598-019-42501-6

**Published:** 2019-04-11

**Authors:** Im-kyung Kim, Yun Sun Lee, Hyung Sun Kim, Seung Myung Dong, Joon Seong Park, Dong Sup Yoon

**Affiliations:** 1 Department of Surgery, Yonsei University College of Medicine, Seoul, Korea; 2 Brain Korea 21 Plus Project for Medical Science, Yonsei University College of Medicine, Seoul, Korea; 3 Interpark Bio Convergence Center, Seoul, Korea

## Abstract

Specific protein 1 (SP1) is associated with aggressive behavior, invasive clinical phenotype and poor clinical outcomes in various cancers. We studied whether SP1 exerts its effect on invasiveness and promotion of the epithelial-mesenchymal transition (EMT) by regulating lysyl oxidase-like 2 (LOXL2) in pancreatic ductal adenocarcinoma (PDAC) cell lines. We showed that silencing of SP1 in MIA Paca-2 cell significantly decreased cell invasion and migration. In MIA Paca-2 cells, silencing of SP1 induced a reduction of LOXL2 expression, whereas LOXL2 silencing did not lead to a decrease in the expression of SP1. Chromatin immunoprecipitation assay demonstrated the binding of SP1 to LOXL2 promoter. Wound healing and transmigration assays also showed that transfection of both SP1 and LOXL2 siRNA induced most significant decrease of cell invasion and migration compared to either SP1 or LOXL2-only silenced cells. Finally, we investigated the prognostic value of SP1 in patients with PDAC and SP1/LOX2 expression was examined by immunochemistry. Univariate and multivariate analyses showed that tumor differentiation and co-expression of SP1 and LOXL2 were independent factors for disease-free survival. In summary, our study demonstrates that SP1 modulates EMT and is involved in tumor invasion and migration of PDAC cells through the regulation of LOXL2.

## Introduction

Pancreatic cancer is the 5^th^ leading cause of cancer-related deaths in Korea and is among the most lethal of all malignancies^1^. Early local invasion and high recurrence rate contributes to the poor prognosis. Therefore, it is important to fully understand the mechanism of early invasion and epithelial-mesenchymal transition (EMT) in pancreatic cancer.

Specific Protein 1 (SP1), identified as a transcription factor for the simian virus 40 major immediate early gene^2,3^, is required for the transcription of housekeeping genes^4^. Importantly, high SP1 levels in various neoplasms are correlated with aggressive behavior, invasive clinical phenotypes, increase recurrence rates, and decreased survival^5–8^, and both SP1 and EMT markers levels are elevated in cancer cells^9^. Also in pancreatic cancer, Hang *et al*. recently reported that SP1 is involved in the angiogenesis and metastasis of pancreatic cancer by upregulating the expression of cyclooxygenase-2^8^.

LOX-like 2 (LOXL2) is a member of the lysyl oxidase (LOX) family, responsible for the stabilization of collagen and elastin fibers in extracellular matrix^10^. Especially, most studies in regards to oncogenic function of LOXL2 have been reported in breast cancers. One research discovered the positive correlation between the onset of EMT and LOXL2 expression, which suggested LOXL2 contributes to the induction of EMT^11^. Likewise, upregulation of LOXL2 is associated with oncogenic stress response and tumorigenesis in pancreatic ductal adenocarcinoma (PDAC)^12^. Additionally, in pancreatic cell lines, LOXL2 activates EMT-like processes, which are associated with invasive and migratory properties^13^.

SP1 has been indicated as one of the transcription factors involved in LOXL2 expression^14^. Therefore, here, we investigated the effect of SP1 on invasiveness and EMT in PDAC cells through LOXL2 regulation and evaluated the clinical impact of SP1 as a prognostic factor in patients with pancreatic cancer.

## Materials and Methods

### Patients

A total of 84 consecutive patients who underwent R0 resection for PDAC from November 2002 to May 2012 at a single tertiary hospital were enrolled in this study. Twenty-two patients with incomplete medical records, lost to follow-up or without tissue samples were excluded, and the data of 62 patients were retrospectively reviewed. Patients were examined for follow-up every three months during the first year, and every six months afterwards. This study was approved by the Institutional Review Board of Gangnam Severance Hospital, Yonsei University, Seoul, Korea (3-2014-0153). Informed consent was obtained from all participants, and all methods were performed in accordance with the relevant guidelines and regulations.

### Immunohistochemical (IHC) Staining

An anti-SP1 antibody (Thermo Fisher Scientific, Cleveland, OH, USA) and an anti-LOXL2 antibody (Abcam, Cambridge, UK) were used to detect the SP1 and LOXL2. ICH staining procedures and intensity categorization have been described in our previous report^13^. Two pathologists without any information of the enrolled patients were participated in the interpretation of IHC.

### Cell culture

We used human PDAC cell lines (MIA Paca-2, PANC-1, AsPC-1, and BxPC-3) obtained from the American Type Culture Collection (ATCC; Manassas, VA, USA), and cells were grown according to the ATCC recommendations.

### SP1 RNA interference

For knockdown of gene expression, the following small interfering RNA (siRNA) were purchased from Bioneer (Daejeon, Korea): siSP1 (sense: 5′–CA GAUACCAGACCUCUUCU-3′, antisense: 5′-AGAAGAGGUCUGGUAUC UG-3′), siLOXL2 (sense: 5′-CAGUCUAUUAUAGUCACAU-3′, antisense: 5′-AUGUGACUAUAAUAGACUG-3′). Transfection was carried out using TransIT® -LT1 (Mirus Bio, Madinon, Wi, USA) or Lipofectamine 2000 (Invitrogen, Carlsbad, CA, USA) according to the manufacturers’ instructions. Cells were harvested and processed 48–72 hours post-transfection.

### Reverse transcription polymerase chain reaction (RT-PCR)

RNA was extracted using TRIzol (Invitrogen), and purified using an RNA extraction kit (Easy-Spin Total RNA extraction kit, Intron, Korea). For RT- PCR, we used a one-step RT-PCR kit (Intron) and a GeneAmp PCR System 9700 (Appied Biosystems, Foster City, CA, USA) according to the manufacturers’ instructions.

The primers used for RT PCR were SP1 (F: 5′-CCATACCCCTTAACCCCG-3′, and R: 5′-GAATTTTCACTAATGTTTCCCACC-3′). The primers of LOXL, Snail, CDH1, L1CAM, and GAPDH were same as our previous report^13^.

### Western blot analysis

The procedure of Western blot analysis was described in our previous report^13^.

### Wound healing assay

For wound healing assays, we used 24-well culture inserts (ibidi, Martinsried, Germany). Cells were transfected and, 24 hours after, were counted: 70 μL of a cell suspension at 3 × 10^5^ cells/mL was seeded on each side of the insert. After 24 hours, the insert was removed to create a wound, and 0.5 mL of growth medium was added. The cells were washed three times with phosphate-buffered saline and incubated for 24 hours. The wounds were then visualized under a microscope (Axio Observer Z1, Zeiss, Jena, Germany).

### Transmigration assay

For the transmigration assays, the upper side of 8.0-μm Transwell^®^ inserts (Corning Costar, NY, USA) was coated with Matrigel (BD Bioscience). After transfection, 1 × 10^5^ of MIA Paca-2 cells were re-suspended in serum-free medium and seeded on the upper chambers. The lower chambers were filled with growth medium. After 24 hours, the cells on the lower surface of the membrane were stained using a Diff-Quick staining kit (Sysmex, Kobe, Japan), and counted under an inverted microscope. Values are expressed as mean cell numbers in five random fields of view (200×).

### Cell proliferation assay

Cells were seeded in a 96-well plate at a 1 × 10^3^ cells/well, 24 hours before the siRNA transfection. siRNA was transfected by following the manufacturers’ instructions. After 48 hour incubation, medium was replaced to fresh medium in a final volume of 100 μl/well. 10 μl of WST (Ez-cytox, Dogen, Korea) was added to each well and incubated for 1 hours. The absorbance of each well was measured at 450 nm using a microplate reader.

### Immunofluorescence assay

MIA Paca-2 and PANC-1 cells were grown on 4 well chamber slides. The cells were washed three times with PBS after SP1 siRNA treatment, and then fixed in 4% paraformaldehyde for 15 min and permeabilized with 0.1% Triton ×100 (Sigma) for 10 min. After three time washing with PBS, incubate cells with 1% BSA in PBST (PBS + 0.1% Tween20) for 30 min. Polyclonal rabbit antibodies against human SP1 (1:200, Abcam, Cambridge, UK), F-actin (1:200, Abcam, Cambridge, UK) were applied overnight at 4 °C. Washed cells were incubated with Secondary Alexa Fluor 555 anti-rabbit IgG (1:1000, Cell signaling) and Alexa Fluor 488 anti-mouse IgG (1:1000, Cell signaling) for 1 hour at room temperature, washed again. Cells were mounted in DAPI Staining Solution (Abcam, Cambridge, UK), and analyzed using confocal microscopy (LSM510, Carl Zeiss, Jena Germany).

### Chromatin Immunoprecipitation(ChIP)

ChIP assays were performed with the EpiTect ChIP Oneday kit (QIAGEN, Hilden, Germany) following the manufacturer’s instructions. A total of 5 × 10^6^ MIA Paca-2 cells were cross-linked with 1% formaldehyde at room temperature for 10 min. Sonication was performed on ice to get 200 to 1,000 bp DNA fragments. Chromatin was pre-cleared with anti-IgG antibody (Santa Cruz Biotechnology) and immunoprecipitated with an anti-SP1 antibody. After reverse cross-linking and DNA isolation and purification, DNA from input (diluted 1:100) or immunoprecipitated samples were assayed by quantitative PCR (qPCR), performed on a LightCycler®480 Real-Time PCR system (Roche, Diagnostics, Mannheim, Germany) using the SYBT Green I Mastermix (Applied Biosystems). The products were then separated by 2% agarose gel electrophoresis. The following primers were used: P1 (−898 to −778, F: 5′-CAACCCTGACCCCAGCCTCT-3′, and R: 5′-GCAAGGAAAGAGAC ACAGCA-3′), P2 (−878 to −758, F: 5′-GTCCTTTACTCCACAGATAGAT GGGT-3′, and R: 5′-GGGACAACTATTTGGCTGCAG-3′), P3 (−759 to −639, F: 5′-CTGCGCCAAATAGTTGTCCC-3′, and R: 5′-AGCAGGGCTTAGAG AGAAACG-3′), P4 (−568 to 448, F: 5′-GGACATCTGGGGGTCCTCT-3′, and R: 5′-GCGGATTTTGCATTTCACTCCA-3′), P5 (−210 to 90, F: 5′-GTTGC CCAGAGCTCACTG-3′, and R: 5′-GGTTACCCTACTGGCCTGG-3′).

### Statistical analysis

All statistical analyses were performed using the SPSS software, version 21.0 (SPSS Inc., Chicago, IL, USA). Categorical variables were evaluated using the chi-square or the Fisher’s exact tests, and continuous variables were analyzed using the Student’s t-test. Survival curves were plotted using the Kaplan-Meier method, and differences in survival time among groups were assessed with the log-rank test. Disease-free survival (DFS) was measured from the date of surgery to the date of recurrence or last follow-up. Univariate and multivariate analyses using the Cox proportional hazards regression method were performed. A *P-value* < 0.05 was considered significant.

## Results

### Expression of SP1 in human PDAC cell lines

**Figure 1:**
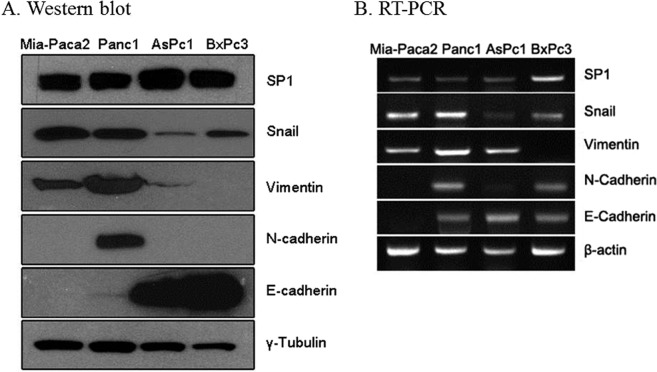
Expression of SP1 and EMT markers in PDAC cell lines. (**A,B**) Western blot analysis (**A**) and RT-PCR (**B**) relative to the expression of SP1 and EMT markers in the indicated cell lines. γ-tubulin (**A**) and β-actin (B) were used for normalization.

Western blotting and RT-PCR were performed in four different PDAC cell lines: MIA Paca-2, PANC-1, AsPC-1, and BxPC-3 cell lines. SP1 was expressed in common at all cell lines, whereas epithelial (E-cadherin) and mesenchymal (Snail, Vimentin, N-cadherin) markers showed varying levels of expression (Fig. 1). MIA Paca-2 and PANC-1 cells had relatively stronger mRNA expression of Snail compared to other cell lines, and MIA Paca-2 showed no expression of N-cadherin. These results were consistent with previous researches^15–17^, which demonstrated heterogenous expression of EMT markers as a reflection of own aggressive and metastatic behavior of each cell line.

### Correlation between SP1 expression and EMT markers in PDAC

**Figure 2:**
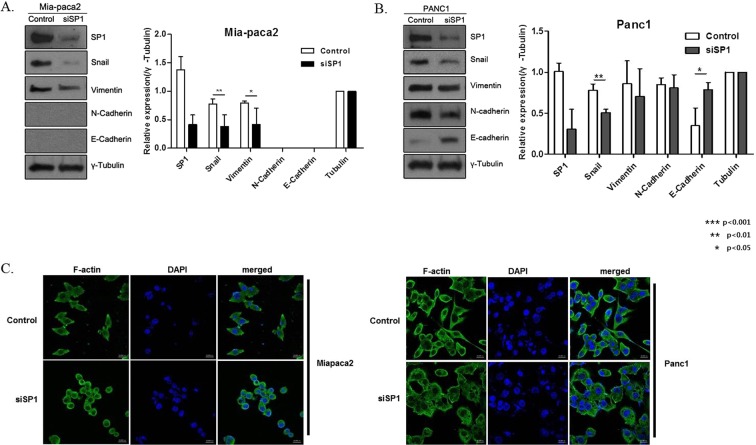
SP1 affects the expression of EMT markers in PDAC cell lines. (**A**) Western blotting and quantification using Image j program of the indicated proteins in MIA Paca-2 cells: silencing of SP1 (siSP1) reduced SP1, snail, and vimentin levels. (**B**) Western blotting and quantification using Imagej program of the indicated proteins in PANC-1 cells: siSP1 reduced SP1 and snail, and increased E-Cadherin. γ-tubulin was used for normalization. (**C**) Immunofluorescence assay to confirm the effect of siSP1 treatment on pancreatic cancer cell morphology. Both cell lines were transformed to epithelial cell shape after siSP1 treatment.

To evaluate the effect of SP1 on the expression of EMT markers, we generated MIA Paca-2 and PANC-1 cells in which SP1 had been silenced. SP1-silenced cells exhibited significant reduction in the expression of Snail, important for EMT. Additionally, in Paca-2-siSP1, the expression of Vimentin was reduced, and in PANC-1-siSP1, the expression of E-cadherin was increased (Fig. 2A,B). We also performed immunofluorescence assay to show the morphological changes after siSP1 treatment. After siSP1 treatment, both cell lines were transformed to epithelial cell shape with exhibiting the cortical actin architecture, and this tendency was more prominent in PANC-1 cells compared to MIA Paca-2 cells (Fig. 2C).

### Effects of SP1 expression on cell invasion and migration

**Figure 3:**
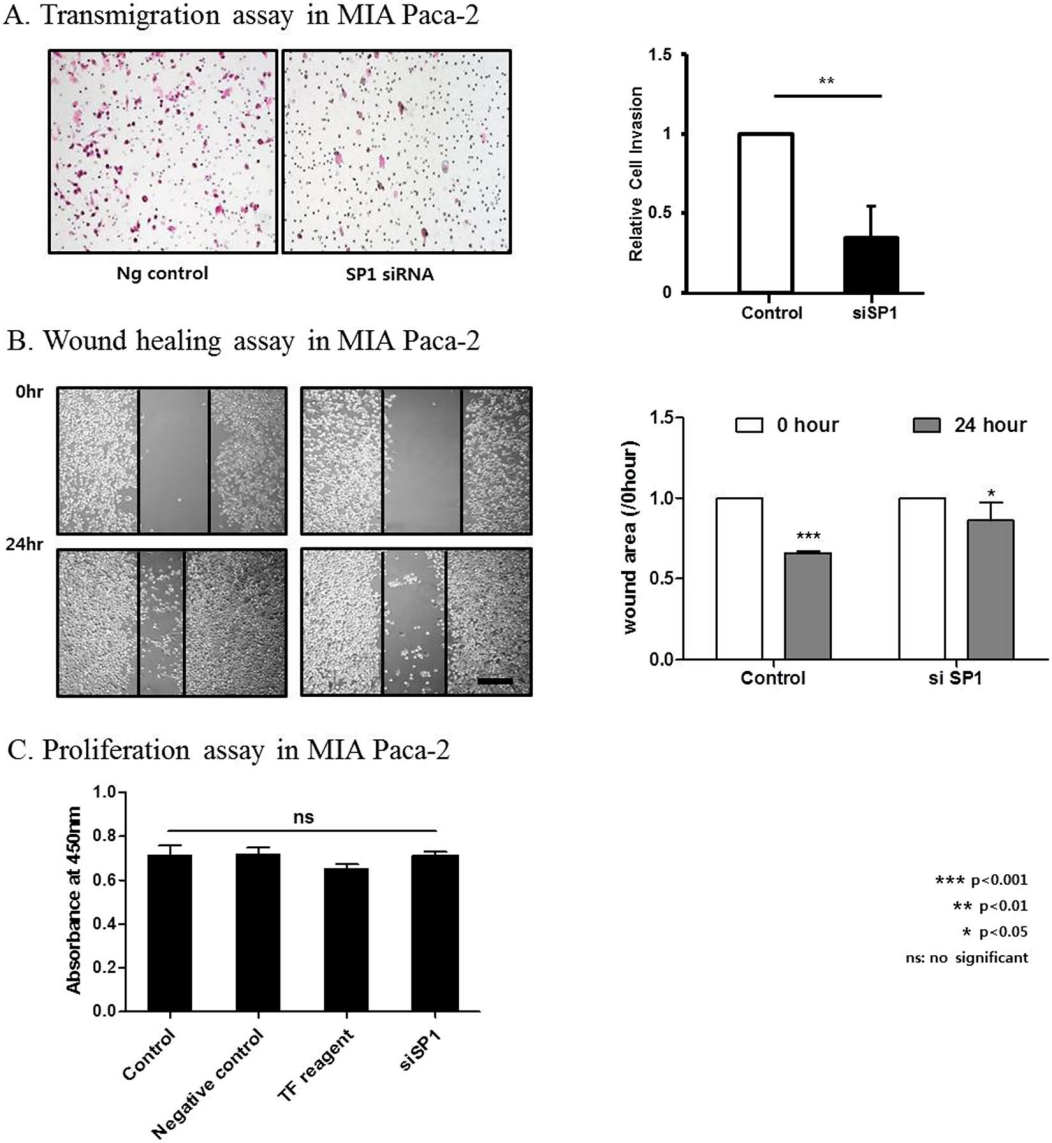
Knockdown of SP1 inhibits the invasion and migration of PDAC cells. (**A**) Transmigration assay in MIA Paca-2 cells transfected with control or SP1 siRNAs: SP1 knockdown significantly inhibits cell invasion. (**B**) Wound healing assay in MIA Paca-2 cells transfected with control or SP1 siRNAs: SP1 knockdown significantly decreased cell migration 24 hours after removal of the insert (see Materials and Methods for details). (**C**) Proliferation assay to compare cell growth rates among control, negative control, transfection reagent and siSP1 treatment groups. There was no significant difference in absorption at 450 nm among the groups.

In transmigration assays, SP1-silenced MIA Paca-2 cells showed a statistically significant decrease of cell invasion (Fig. 3A). Moreover, in wound healing assays, SP1 silencing significantly decreased cell migration compared to control cells (Fig. 3B). On the other hands, the proliferation assay showed no significant cell growth after siSP1 treatment compared to negative controls (Fig. 3C).

### EMT regulation by SP1-LOXL2 axis

**Figure 4:**
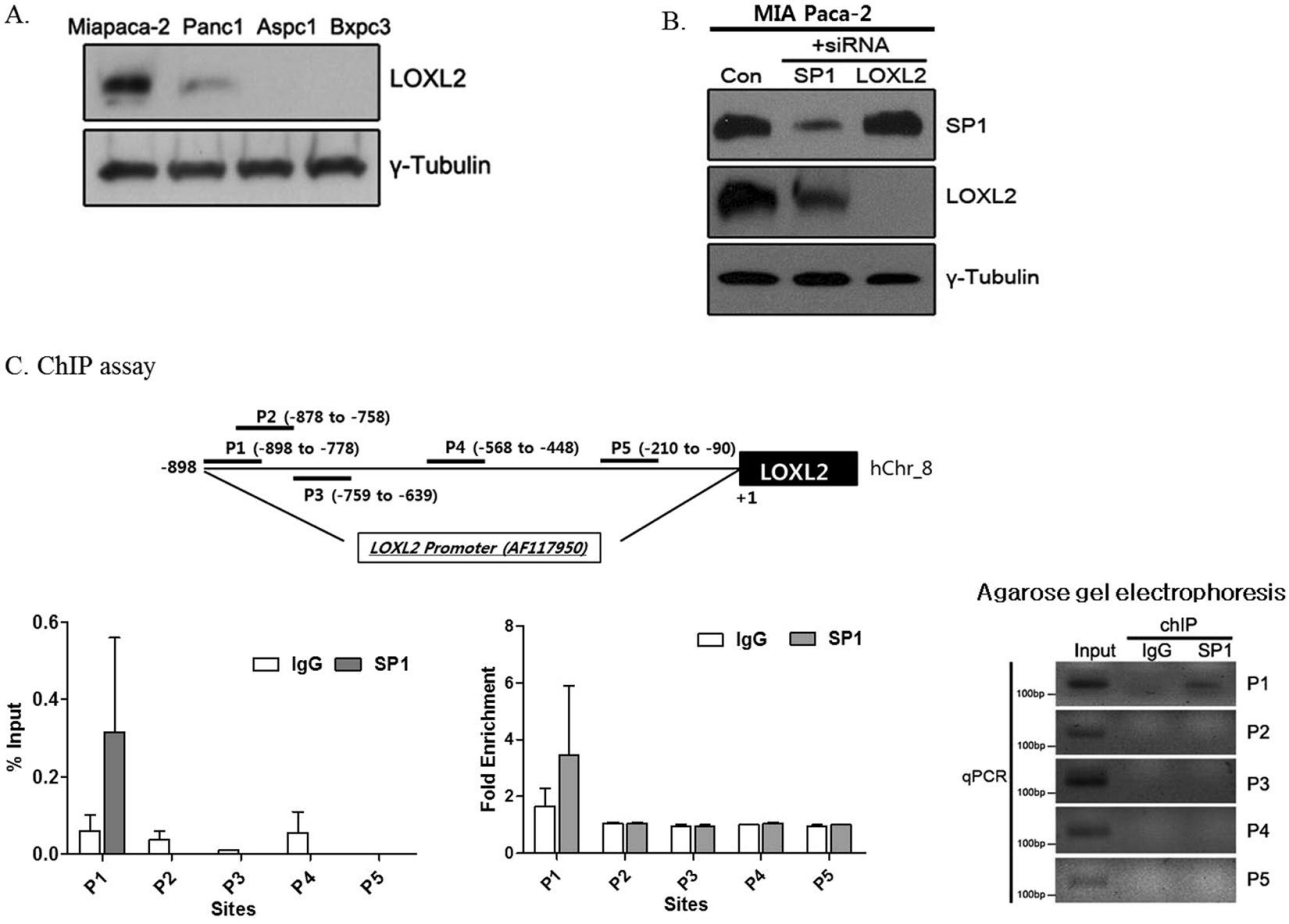
SP1 regulates the expression of EMT-related factor LOXL2. (**A**) LOXL2 expression in PDAC cell lines. (**B**) Western blot analysis after silencing LOXL2 or SP1 in MIA Paca-2 cells: SP1 silencing induced a reduction of LOXL-2 expression, whereas LOXL-2 silencing did not decrease the expression of SP1. (**C**) ChIP assay. The scheme indicates LOXL2 promoter and the regions recognized by the primers used in the qPCR assays. These assays show that SP1 binds to the P1 region of LOXL2 promoter.

Higher LOXL2 expression is associated with the invasiveness of PDAC and the lower survival rate of patients. As demonstrated in Fig. 4A, LOXL-2 was detected in MIA Paca-2 and PANC-1 cells, in agreement with our previous study4. In MIA Paca-2, SP1 silencing induced a reduction of LOXL-2 expression, whereas LOXL-2 silencing did not affect SP1 levels (Fig. 4B). Additionally, to confirm the transcriptional regulation of LOXL2 by SP1, ChIP assay with qPCR was performed. We identified the promoter region of LOXL2 containing one of the predicted SP1 binding sites described in a recent research by Jourdan-Le Saux C *et al*.^14^ (Fig. 4C). Corresponding to ChIP assay, the agarose gel electrophoresis also demonstrated SP1 binding^18^ to P1 site.

**Figure 5:**
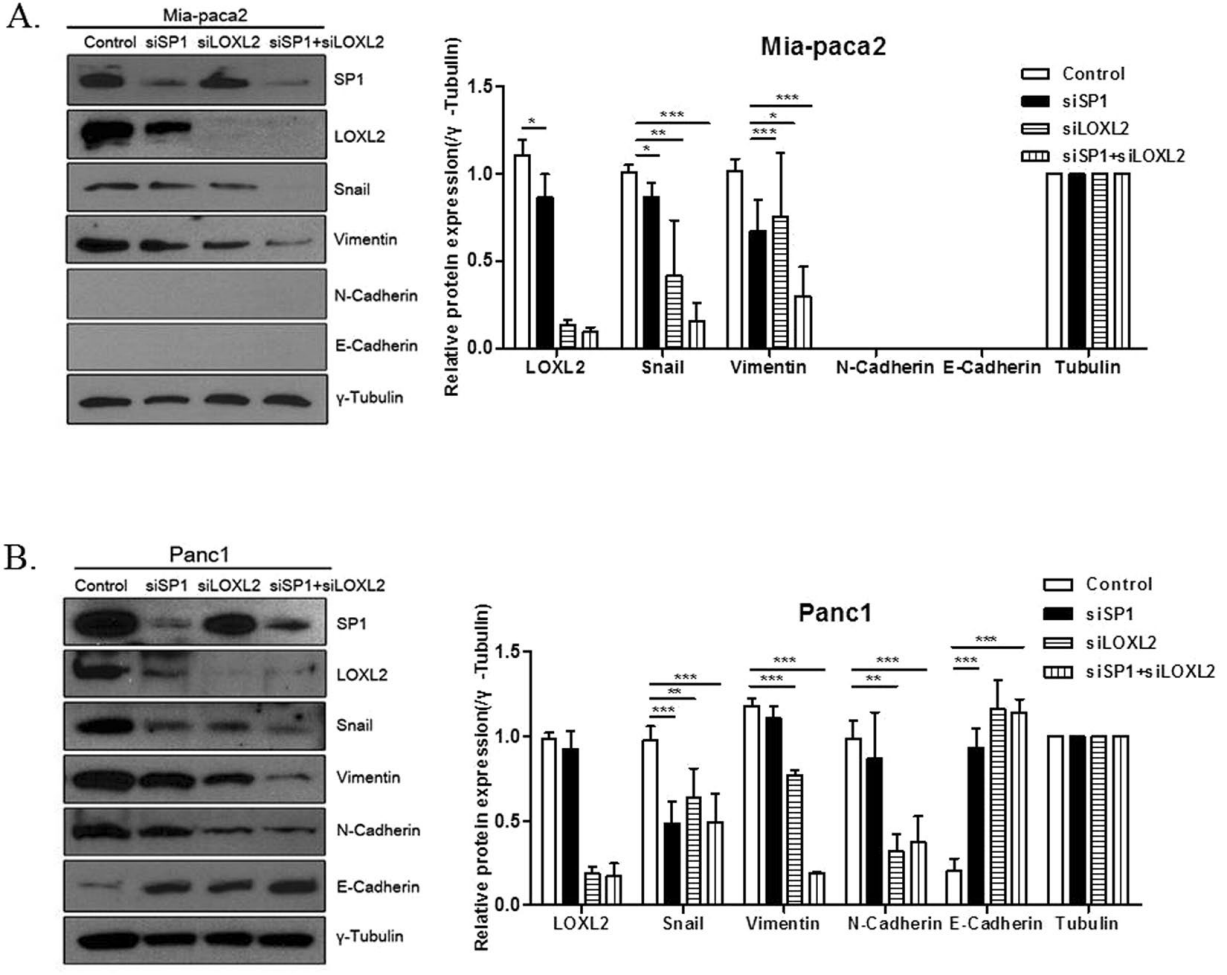
The effect of SP1 and LOXL2 silencing on EMT. (**A,B**) Western blotting and quantification using Image j program in MIA Paca-2 and PANC-1; silencing of SP1 and LOXL2 induced a remarkable decrease of EMT markers as well as silencing of LOXL2 alone.

**Figure 6:**
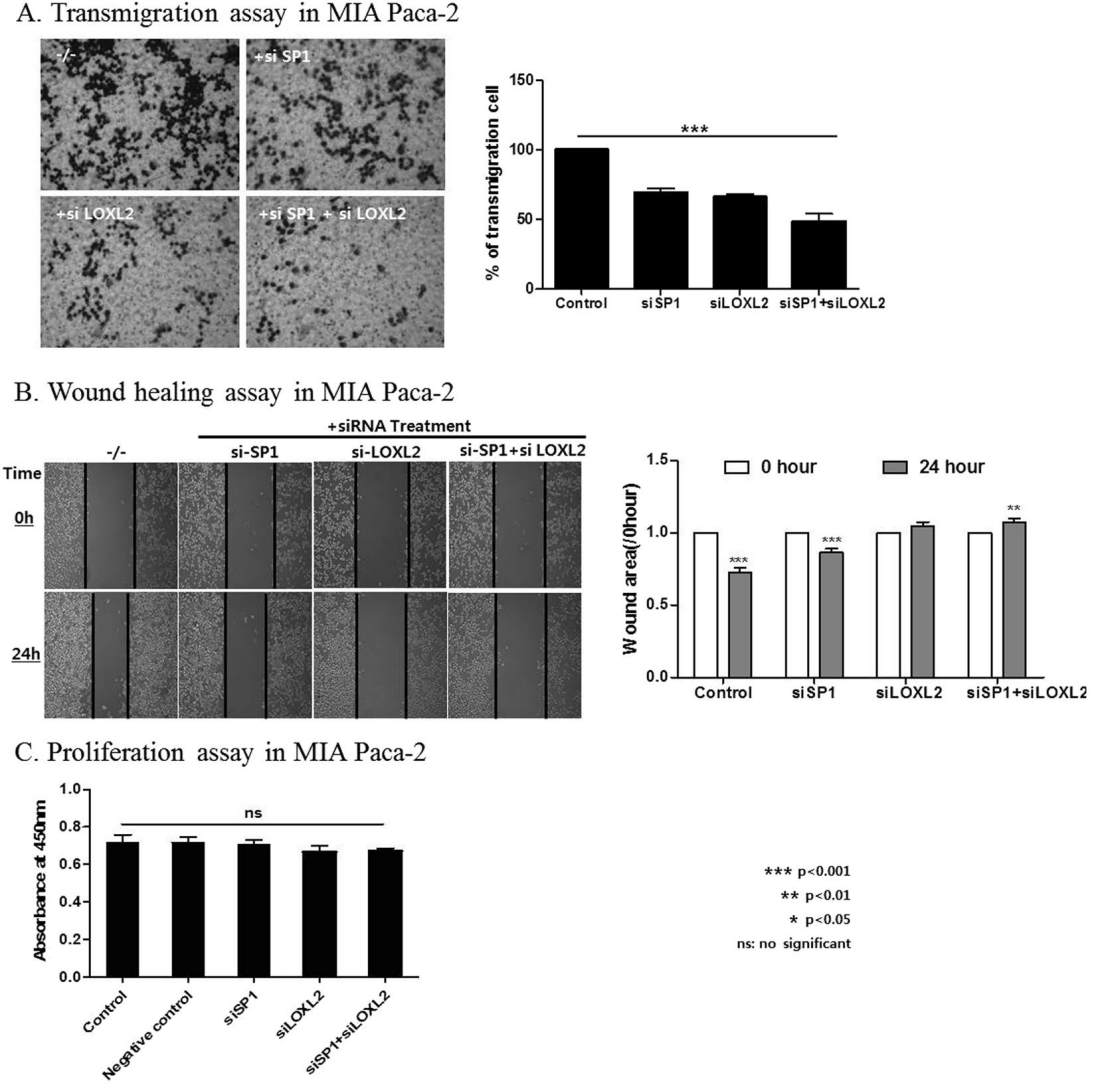
SP1 regulates EMT of PDAC cells through LOXL2. (**A**) Transmigration assay in MIA Paca-2; transfection of both SP1/LOXL2 siRNA induced the most significant decrease of cell invasion compared to SP1-only silenced cells. (**B**) Wound healing assay in MIA Paca-2; transfection of both SP1/LOXL2 siRNA decreased cell migration more than transfection of SPI siRNA. (**C**) Proliferation assay to compare cell growth rates among control, negative control, transfection reagent and siSP1 treatment groups. There was no significant difference in absorption at 450 nm among the groups.

To investigate how SP1 regulates EMT through LOXL2, we performed additional silencing experiments. In MIA Paca-2 and PANC-1 cells, silencing of both factors induced a remarkable decrease of EMT marker levels, higher than that obtained upon silencing of either SP1 or LOXL2 (Fig. 5A,B). Additionally, transmigration and wound healing assays showed the greatest decline in cell migration when both factors were silenced (Fig. 6A,B). Again, the proliferation assay showed no significant cell growth after siSP1 treatment compared to negative controls (Fig. 6C).

### SP1 and LOXL2 expression in human PDAC samples

**Figure 7:**
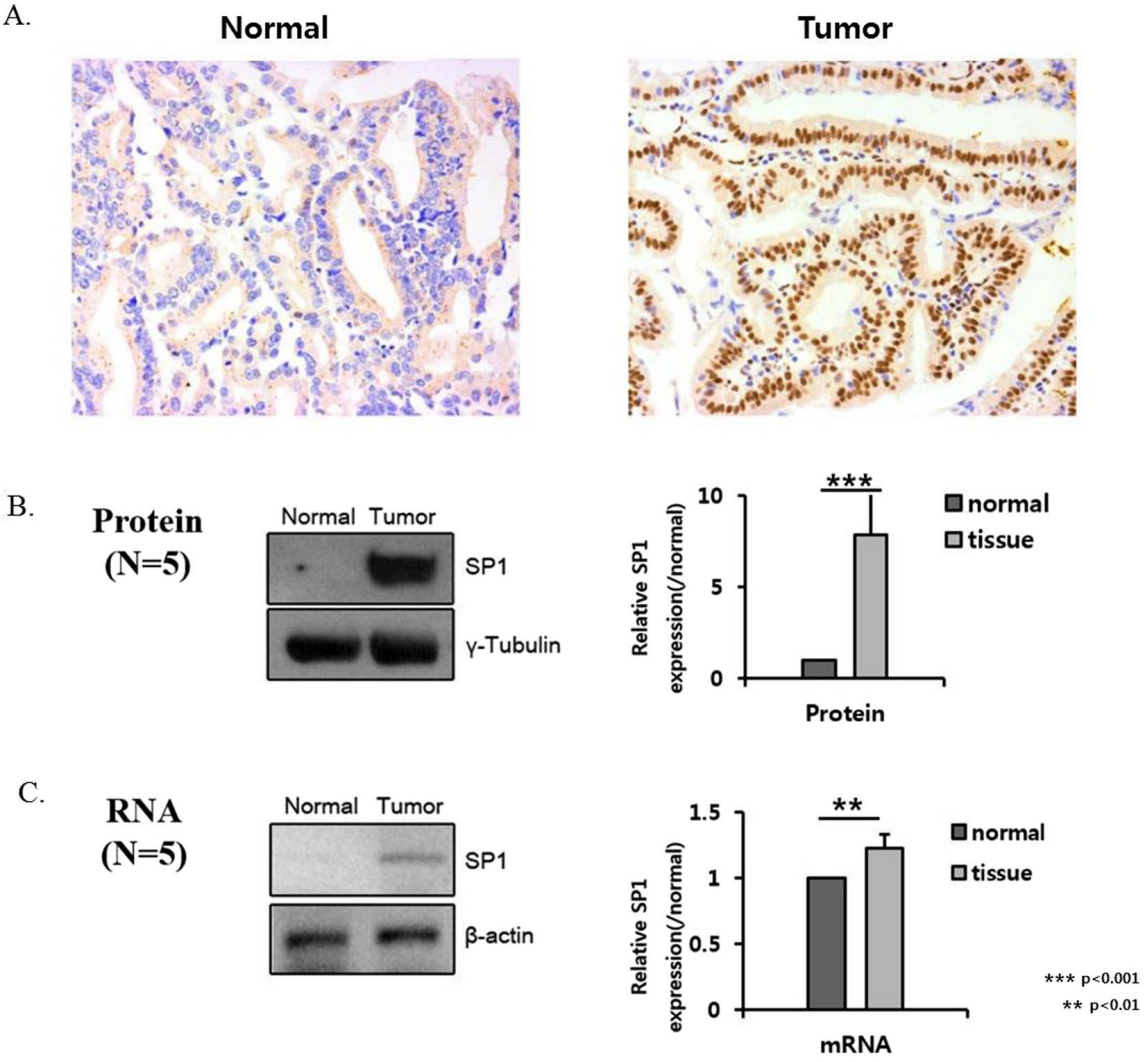
Expression of SP1 in human tissues. (**A**) Immunohistochemistry of SP1 in normal and cancer tissue. (**B**) Western blot analysis of SP1 protein expression in normal and tumor tissues (N = 5). The graph indicates the quantification of the results. (**C**) RT-PCR analysis of SP1 mRNA expression in normal and tumor tissues (N = 5). The graph indicates the quantification of the results.

Next, we investigated SP1 and LOXL2 expression in PDAC samples from 62 patients. As described in Fig. 7, in PDAC tissue, the overexpression of SP1 was observed, whereas the normal pancreatic tissue did not express SP1 by IHC staining, western blotting and RT-PCR. Patient characteristics according to the expression of SP1/LOXL2 evaluated by IHC staining are described in Table 1. There was no significant difference in cell differentiation, tumor size, invasion depth or lymph node metastases among patient groups divided according to the expression of SP1/LOXL2.

**Table 1:** Patient characteristics according to the expression of SP1 and LOXL2.

Factors	N = 62	SP1			LOXL2		
		Negative (n = 38)	Positive (n = 24)	*P*	Negative (n = 14)	Positive (n = 48)	*P*
Sex				0.434			0.368
Male	28	19 (50.0%)	9 (37.5%)		8 (57.1%)	20 (41.7%)	
Female	34	19 (50.0%)	15 (62.5%)		6 (42.9%)	28 (58.3%)	
Age				0.311			0.7
≤60	11	5 (13.2%)	6 (25.0%)		3 (21.4%)	8 (16.7%)	
>60	51	33 (86.8%)	18 (75.0%)		11 (78.6%)	40 (83.3%)	
Operations				1			0.315
PD	44	27 (71.1%)	17 (70.8%)		8 (57.1%)	36 (75.0%)	
Others	15	11 (28.9%)	7 (29.2%)		6 (42.9%)	12 (25.0%)	
Differentiation				0.505			1
Well	11	8 (21.1%)	3 (12.5%)		2 (14.3%)	9 (18.8%)	
Mod. to Poor	51	30 (78.9%)	21 (87.5%)		12 (85.7%)	39 (81.3%)	
Tumor size (mean ± SD, cm)		3.20 ± 1.32	3.18 ± 1.36	0.955	3.26 ± 1.03	3.16 ± 1.40	0.805
Invasion depth				0.736			1
T1/2	51	32 (84.2%)	19 (79.2%)		12 (85.7%)	39 (81.3%)	
T3/4	11	6 (15.8%)	5 (20.8%)		2 (14.3%)	9 (18.8%)	
LN metastasis				1			0.519
Negative	19	12 (31.6%)	7 (29.2%)		3 (21.4%)	16 (33.3%)	
Positive	43	26 (68.4%)	17 (70.8%)		11 (78.6%)	32 (66.7%)	
PNI				0.764			1
Negative	15	10 (26.3%)	5 (20.8%)		3 (21.4%)	12 (25.0%)	
Positive	47	28 (73.7%)	19 (79.2%)		11 (78.6%)	36 (75.0%)	
LVI				0.434			1
Negative	31	21 (55.3%)	10 (41.7%)		7 (50.0%)	24 (50.0%)	
Positive	31	17 (44.7%)	14 (58.3%)		7 (50.0%)	24 (50.0%)	

Abbreviation: N, number; PD, pancreaticoduodenectomy; Mod., moderate; LN, lymph node; PNI, perineural invasion; LVI, lymphovascular invasion.

**Figure 8:**
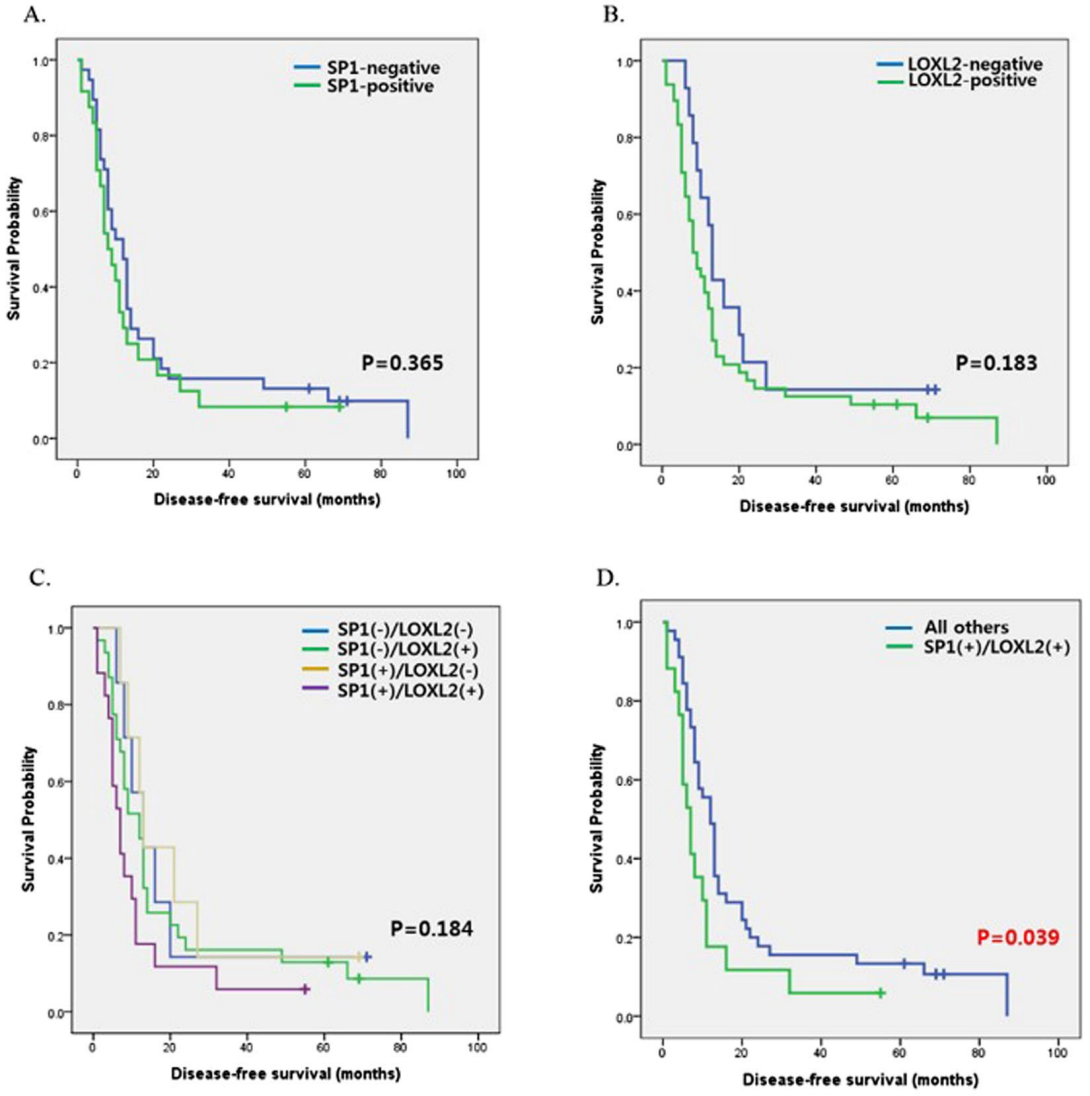
Disease-free survival analyses according to the expression of SP1 and LOXL2. (**A,B**) 5-year DFS curves according to the expression of SP1 or LOXL2: no significant differences were found according to the expression of SP1 or LOXL2. (**C**) 5-year DFS curves according to the co-expression of two factors: there was no significant difference in 5-year DFS rates among the four indicated groups. (**D**) 5-year DFS curves according to the expression of both SP1 and LOXL2 in the SP1(+)/LOXL2(+) vs. the SP1(−)/LOXL2(−), SP1(−)/LOXL2(+), and SP1(+)/LOXL2(−) groups. The group with SP1(+)/LOXL2(+) showed significantly lower DFS compared to all others.

However, multivariate analyses indicated that tumor differentiation and high expression of both SP1 and LOXL2 were independent poor prognostic factors for DFS (Table 2). Additionally, Kaplan Meier survival analyses showed that the patients with high expression of both SP1 and LOXL2 had lower DFS compared to other groups with clinical significance (Fig. 8).

**Table 2:** Univariate and multivariate analyses of the relationship between DFS and clinicopathologic variables by Cox regression hazard model.

Factors	N	DFS	Univariate analysis			Multivariate analysis		
		median (range)	HR	95% CI	*P*	HR	95% CI	*P*
**Differentiation**								
Well	11	16.1 (5.8–87.4)	1	1.365–6.817	**0.007**	1	1.528–7.899	**0.003**
Mod. to Poor	51	8.3 (1.1–71.4)	3.05			3.474		
**Invasion depth**								
T1/2	51	10.3 (1.1–87.4)	1	0.653–2.592	0.455			
T3/4	11	11.8 (3.5–69.7)	1.301					
**LN metastasis**								
Negative	19	8.3 (1.1–69.5)	1	0.483–1.512	0.59			
Positive	43	11.0 (1.5–87.4)	0.855					
**PNI**								
Negative	15	11.3 (1.1–71.4)	1	0.501–1.741	0.83			
Positive	47	9.2 (3.5–87.4)	1.071					
**LVI**								
Negative	31	12.7 (1.1–69.7)	1	0.610–1.749	0.903			
Positive	31	9.4 (1.5–87.4)	1.033					
**SP1 status**								
Negative	38	12.7 (1.1–87.4)	1	0.742–2.176	0.384			
Positive	24	8.1 (1.5–69.7)	1.27					
**LOXL2 status**								
Negative	14	13.2 (7.0–71.4)	1	0.799–2.875	0.203			
Positive	48	8.3 (1.1–87.4)	1.516					
**SP1/LOXL2 status**								
All others	45	12.7 (1.1–87.4)	1	1.001–3.244	**0.039**	1	1.170–3.953	**0.014**
SP1(+)/LOXL2(+)	17	7.1 (1.5–56.0)	1.802			2.151		

Abbreviation: N, number; DFS, disease-free survival; Mod., moderate; LN, lymph node; PNI, perineural invasion; LVI, lymphovascular invasion.

## Discussion

PDAC is characterized by extensive stromal and matrix deposition (*demoplasia)*, which generates high levels of stress in the tumors and compression of the vasculature, and promotes a hypoxic, nutrient-poor environments^18^. These conditions cause endoplasmic reticulum(ER) stress, which, in turn, initiates the unfolded or incorrectly folded protein response^19–21^.

SP1 is a highly regulated transcription factor that plays a critical role for the regulation of genes involved in cancer progression^22^. Earlier studies have demonstrated SP1 overexpression in various cancers including sarcoma, and adenocarcinomas in colon or stomach^5–7,23–25^, whereas minimal or no expression of SP1 was detected in normal differentiated cells. Similarly, SP1 is overexpressed in pancreatic cancer^8,9,19^. It has been reported that downregulation of SP1 interferes with recovery from ER stress, and activation of unfolded protein response in PDAC^19^.

On the other hand, the overexpression of LOXL2 in PDAC cells induces EMT, cell migration and invasion^13^. Additionally, LOXL2 is one of the most specifically and highly expressed genes in pancreatic cancer. Therefore, several groups have investigated the mechanisms that cause LOXL2 upregulation. One recent study reported that the promoter of LOXL2 contains binding sites for several transcription factors including SP1^14^. In this study, we found that SP1 binds to LOXL2 promoter and regulates the expression of EMT markers.

Our clinical data demonstrated that the overexpression of SP1 and LOXL2 correlates with poor prognosis in patients with pancreatic cancer. However, the overexpression of either SP1 or LOXL2 did not significantly correlate with poor clinical outcomes including lymph node metastasis, depth of invasion, or lymphovascular invasion. These unexpected results might be driven from the basic issue that SP1 regulates the expression of heterogenous target proteins^9^. Therefore, the signaling pathway through SP1-LOXL2 axis is not the only one contributing to tumor invasion and progression in pancreatic cancer.

Moreover, as only tumor core tissue was used in the experiments, this study did not reflect the characteristics of tumor microenvironment. Kasashima *et al*.^26^ demonstrated that LOXL2 expression in stromal cells, not in cancer cells, was associated with poor prognosis in gastric cancer. Considering the nature of pancreatic cancer that produces favorable microenvironment for cancer progression, further additional experiments using tumor stroma or invasion border should have been performed. However, in this study, we could not perform experiments using tumor stroma or invasion border owing to the lack of tissue sample.

Nevertheless, this study indicates that SP1 may serve as a therapeutic target for selected patients with pancreatic cancer, especially if silenced together with LOXL2. However, additional studies of SP1 function and mechanism of action are required prior to practical use of SP1 for pancreatic cancer treatment.

## Conclusion

Our study demonstrated that SP1 modulates EMT and is involved in invasion and migration of PDAC cells by regulating LOXL2. In clinical data, the co-overexpression of SP1 and LOXL2 significantly correlates with poor prognosis in patients with pancreatic cancer.
